# Insulin production in the retina drives autocrine signalling and metabolism reprogramming of the ARPE-19, a retinal pigment epithelium cellular model

**DOI:** 10.1007/s00018-026-06222-0

**Published:** 2026-05-01

**Authors:** Alessandra Puddu, Matilde Balbi, Silvia Ravera, Isabella Panfoli, Davide Maggi

**Affiliations:** 1 Department of Internal Medicine and Medical Specialties, University of Genova, Genova, Italy; 2 Department of Experimental Medicine, University of Genoa, Genova, Italy; 3 IRCCS Azienda Ospedaliera Metropolitana, Genova, Italy; 4 Department of Pharmacy—(DIFAR), University of Genoa, Genova, Italy

**Keywords:** Retinal pigment epithelium, Phagocytosis of photoreceptor outer segment, Retinal metabolism, Local insulin production, AKT signalling

## Abstract

The retinal pigment epithelium (RPE) plays a pivotal role in retinal homeostasis and energy metabolism. A recent study demonstrates that RPE cells release insulin in response to photoreceptor outer segment (POS) phagocytosis and starvation conditions. However, the downstream signalling pathway of this local insulin production has not yet been identified. Therefore, using the ARPE-19 cell line as an in vitro model of human RPE, we have investigated insulin signalling in basal conditions and after rod OS phagocytosis. Our data show that ARPE-19 cells express key pancreatic β-cell markers, including the transcription factor Pancreatic and Duodenal Homeobox-1 (PDX-1), which translocates to the nucleus in response to phagocytosis, and prohormone convertase 1/3 (PC1/3). In addition, ARPE-19 cells synthesize and secrete insulin already in basal conditions, increasing their release after phagocytosis. The RPE-secreted insulin acts in an autocrine manner, activating the canonical insulin signalling pathway and leading to increased phosphorylation of insulin receptor (IR), insulin receptor substrate-1 (IRS-1), and AKT. An upregulation of the insulin-responsive glucose transporter GLUT4 and increased glucose uptake was also observed, fueling the ARPE-19 cells’ oxidative energy metabolism, incrementing the oxidative phosphorylation activity, probably to sustain the high energy demand associated with phagocytosis. At the same time, a decrease in lactate release has been observed. These features may have important implications for understanding retinal energy metabolism and developing novel therapeutic strategies for retinal neurodegenerative diseases.

**Graphical Abstract:**

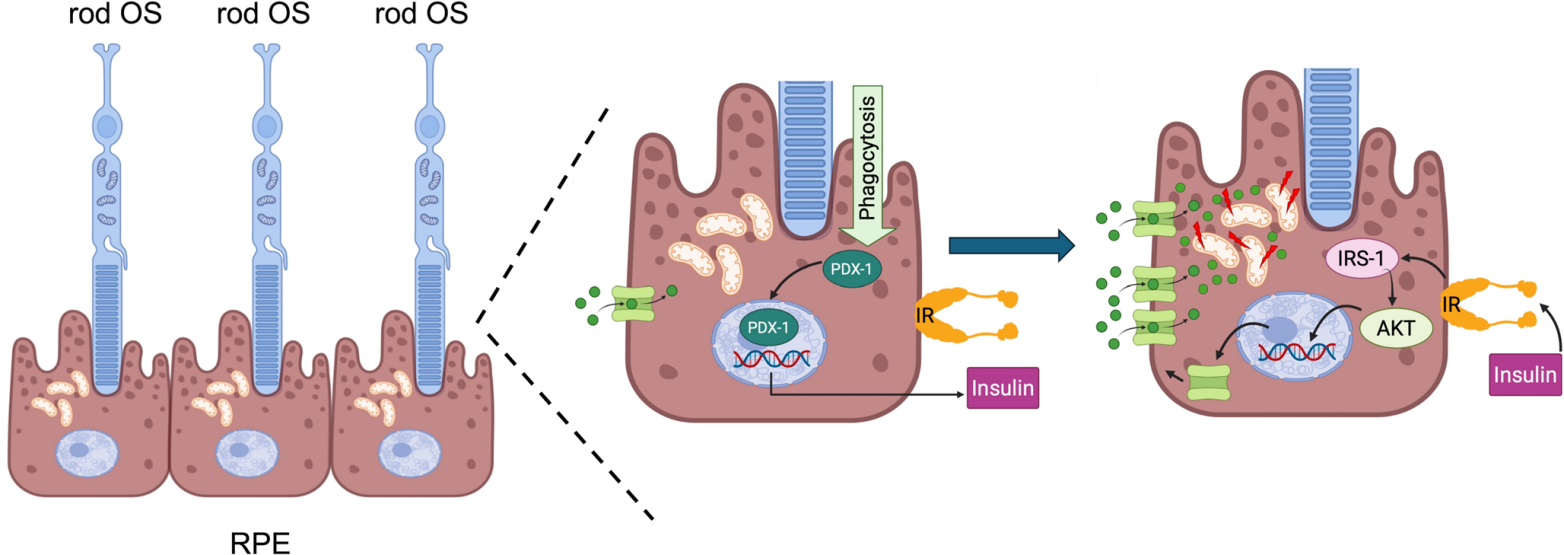

**Supplementary Information:**

The online version contains supplementary material available at 10.1007/s00018-026-06222-0.

## Introduction

The retina is one of the most metabolically active tissues, characterized by a high glucose and oxygen demand [1]. In this context, the retinal pigment epithelium (RPE), a monolayer of highly polarized cells that interact with photoreceptor cells and Bruch’s membrane, that form the outer blood retinal barrier, plays an essential role in maintaining visual function and ensuring retinal homeostasis [2] since its dysfunction can compromise the function and viability of the other retinal cells [3, 4]. In detail, a single RPE cell interacts with approximately 30 photoreceptors, efficiently regenerating 11-cis retinal, and clearing the shed photoreceptor outer segments (POS) to prevent debris accumulation and maintain retinal homeostasis [5, 6]. In addition, RPE supports photoreceptor homeostasis by supplying essential nutrients, including glucose transported from the choroid to the subretinal space via GLUT1on both the apical and basolateral membranes of polarized RPE cells [7–9]. From a metabolic point of view, RPE cells are principally sustained by oxidative phosphorylation (OxPhos) [10]. This high oxidative metabolism is sustained by the RPE’s ability to adjust its metabolic substrate dependence to meet energy demands and optimize substrate utilization [7, 11]. For example, since RPE engulfs and digests the POS damaged by light, efficiently converting fatty acids derived from them into acetyl-CoA by β-oxidation to sustain its energy oxidative metabolism [12]. In addition, RPE OxPhos is supplied by the lactate produced by photoreceptors [13, 14]. Although the metabolic crosstalk with POS provides essential metabolites to RPE cells, and most of the glucose reaching the retina is taken up by photoreceptors, the RPE also metabolizes glucose to support its functions. For example, RPE cells catabolize glucose to produce precursors for the pentose phosphate pathway or to be stored as glycogen [15].

It is well known that glucose uptake in muscle and adipose tissue is regulated by insulin, a small hormone produced by the pancreatic beta cells [16]. However, despite the abundant expression of insulin receptor (IR) in retinal cells, mainly on photoreceptors [17], and the role of altered insulin signal in retinal disease [18, 19], the retina has been considered ‘insensitive’ to systemic insulin [20]. Indeed, injection of insulin into the portal vein of rats leads to phosphorylation of IR in muscle but not in the retina [20]. Furthermore, insulin protein levels and IR phosphorylation in the retina are not affected by nutrient ingestion [21]. These findings are more evident during starvation, when the concentration of insulin in the blood significantly decreases while the concentration of insulin in the retina remains unchanged [22]. This different behavior seems to depend on the slowdown of systemic insulin transport by the blood-retina barrier, creating a more stable, steady-state level that is less prone to the rapid fluctuations seen in other tissues like muscle [23]. In addition, recently, Etchegaray et al. report that the RPE is a site of local insulin production, especially during starvation and following the phagocytosis of damaged photoreceptor outer segments [24, 25]. On the other hand, Jones et al. had already observed that genes for Ins1 and Ins2 were expressed in the retina [26].

Therefore, in this study, we investigated the ability of a RPE cell model, ARPE-19 cells, to produce and release insulin following the phagocytosis of rod outer segments (OS). We evaluated the autocrine effects of the hormone on both cellular signalling and energy metabolism. The data show that ARPE-19 cells respond to insulin by activating IR signalling and by increasing pyruvate-supported oxidative phosphorylation.

## Materials and methods

### Cell line and culture conditions

The human RPE cell line ARPE-19 (passages 24 to 28, from American Type Culture Collection, Manassas, VA, USA) was grown in DMEM/F12 1:1 medium (Euroclone, Milano, Italy) supplemented with 10% fetal bovine serum and 2 mM glutamine (Euroclone, Milano, Italy) at 37 °C in 5% CO_2_ up to confluence. Afterward, cells were seeded in multi-well plates at a density of 2 × 10^4^ cells/cm^2^, grown to confluence, and cultured for 9 days in medium containing 3% FBS to achieve polarization [27, 28]. The proper differentiation of ARPE-19 cells is shown in Supplementary Fig. 1.

### Rod outer segment isolation

Rod OS were isolated from retinas of freshly enucleated bovine eyes obtained from a local slaughterhouse, following a protocol optimized for maximal recovery [29]. All procedures were conducted under dim red illumination to minimize light exposure. Briefly, eyecups, after removal of the vitreous body and lens, were filled with Mammalian Ringer’s solution (0.157 M NaCl, 5 mM KCl, 7 mM Na₂HPO₄, 8 mM NaH₂PO₄, 0.5 mM MgCl₂, 2 mM CaCl₂; pH 6.9) supplemented with a protease inhibitor cocktail (Sigma–Aldrich, St. Louis, MO, USA) and ampicillin (50 µg/mL) and incubated for 10 min. The detached retinas were then gently separated from the optic nerve. Rod OS were subsequently purified by continuous sucrose/Ficoll gradient centrifugation in the presence of protease inhibitors and ampicillin, as previously described [30]. Rod OS suspensions were maintained in darkness until the addition to ARPE-19 cells, and their protein concentration was estimated by Bradford methods [31].

### Evaluation of insulin secretion by ARPE-19 cells

To evaluate the insulin secretion after rod OS phagocytosis, ARPE-19 cells were seeded in a 24-well plate and incubated with 4 µg of total protein of rod OS per cm² cell growth area for 4 h at 37 °C. The amount of rod OS was chosen based on previous literature data [32–34]. After incubation, the medium containing non-ingested rod OS was collected, and the cells were extensively washed with PBS to remove unbound rod OS. Media containing non-digested OS were centrifuged, and the supernatants were stored at − 80 °C until used for the detection of insulin. As control, a batch of ARPE-19 cells was incubated in culture medium w/o rod OS. To verify that ARPE-19 cells phagocytosed rod OS, cells were collected after 4 h of photoreceptor incubation and extensive washing to remove non-internalized material and analyzed by Western blot (described below) to assess the presence of rhodopsin, a specific marker of photoreceptors that is not expressed in ARPE-19 cells unless rod OS have been internalized. The data are reported in Supplementary Fig. 2.

In addition, to study insulin secretion in response to glucose and membrane depolarization, ARPE-19 cells were preincubated 1 h in Kreb’s Ringer Bicarbonate buffer (KRB, containing 118.5 mM NaCl, 2.54 mM CaCl_2_, 1.19 mM KH_2_PO_4_, 4.75 mM KCl, 25 mM NaHCO_3_, 1.19 mM MgSO_4_, 10 mM HEPES, and 0.1% BSA, pH 7.4), then cells were challenged for 10 min either with KRB containing 16.7 mM glucose (HG) or 40 mM KCl. Supernatants were collected and stored at − 80 °C until the insulin determination was performed.

Insulin was evaluated using Lumit^®^ Insulin Immunoassay Kit (Promega, Madison, WI, USA). Secretion was normalized to the protein content of the corresponding cell lysate. Results were shown as pg of insulin release in medium vs. mg protein of the corresponding cell sample lysate.

### Cell lysis and subcellular fractionation

At the end of the experiments on insulin release, a batch of ARPE-19 cells was immediately lysed in RIPA buffer supplemented with protease and phosphatase inhibitor cocktails. Another set of cells was processed for subcellular fractionation using the Subcellular Protein Fractionation Kit (Pierce Biotechnology, Rockford, IL, USA) according to the manufacturer’s instructions. Briefly, various cellular compartments were isolated by sequential addition of different extraction buffers to the cell pellet. Each subcellular fraction was collected after centrifugation and stored at − 80 °C. Cytosolic, nuclear soluble, and chromatin-bound protein extracts obtained from each experimental condition were used for immunoblotting analysis. The protein concentration of each sample was determined using the BCA Protein Assay Kit (Pierce Biotechnology, Rockford, IL, USA). The expression of GAPDH, H2AC2, and H3 as markers of the cytoplasmic, nuclear soluble, and chromatin-bound fractions, respectively, was evaluated by western blot analyses (described below) to verify the quality of sub-fractioning (Data are reported in Supplementary Fig. 3).

### Intracellular insulin signalling

To investigate insulin signalling in RPE, ARPE-19 cells were serum-starved overnight, then exposed for 5 min to 100 nM recombinant human Insulin (INS) or to the KRB collected after stimulation of insulin secretion with high concentration of KCl (KRB KCl). After treatment, the cells were lysed in RIPA buffer supplemented with protease and phosphatase inhibitor cocktails (all from Pierce Biotechnology, Rockford, IL, USA), and protein concentration was determined using the BCA protein assay Kit (Pierce Biotechnology, Rockford, IL, USA).

### Western blot analyses

Fifteen micrograms of total cell lysate or subcellular fractions were separated through denaturing electrophoresis (SDS-PAGE) on 4–20% gradient gels (Life Technologies Italia, Milan, Italy) and transferred onto nitrocellulose using the iBlot system (Life Technologies Italia, Milan, Italy). Filters were blocked in Protein Free T20 Blocking Buffer (Pierce Biotechnology, Rockford, IL, USA) and incubated overnight at 4 °C with the following primary specific antibodies: Insulin (cat A19066), PDX-1 (Pancreatic and Duodenal Homeobox-1, cat A10173), GLUT1 (cat A6982) and GLUT4 (cat A7637) from ABclonal Technology (Woburn, MA, USA); GAPDH (14C10, cat. 2118), phAKT (Ser473, D9E, cat. 4060), phIRS-1 (Tyr895, cat. 3070), phIR (Tyr1345, 14A4, cat 3026), H3 (D1H2, cat. 4499), HDAC2 (3F3, cat. 5113), Rhodopsin (cat. 8710), and β-ACT (8H10D10 cat. 3700) from Cell Signalling Technology, Beverly, MA, USA; and Prohormone Convertase 1/3 (cat. AB10553) from Merck (Darmstadt, Germany). Secondary specific horseradish peroxidase-linked antibodies were added for 1 h at room temperature. The bound antibodies were detected using the enhanced chemiluminescence lighting system (LiteAblot EXTEND, Euroclone, Milan, Italy), according to the manufacturer’s instructions. Each membrane was stripped (Restore PLUS Western blot Stripping Buffer, Pierce Biotechnology, Rockford, IL, USA) and probed for β-actin (Cell Signalling Technology, Beverly, MA, USA) to verify equal protein loading. Bands of interest were quantified by densitometry using the Alliance 1D software. The results were expressed as a percentage of no-treated sample (defined as 100%).

### Evaluation of glucose uptake and lactate release

To investigate whether phagocytosis-dependent insulin release by ARPE-19 cells could affect glucose uptake and lactate release, cells were extensively washed with PBS after POS phagocytosis to remove non-ingested POS and then incubated for 48 h in DMEM/F12 containing 3% FBS, allowing sufficient time to assess any potential change in glucose consumption and lactate release in the medium. As a positive control, 10 nM insulin was added to ARPE-19 cells not incubated with rod OS.

Glucose consumption was calculated by subtracting the glucose content in the medium after 48 h of incubation with ARPE-19 cells from the glucose content in the medium before incubation.

Glucose level in the culture medium was determined by monitoring the reduction of NADP⁺ at 340 nm. For this assay, 5 µL of culture medium was added to a reaction mixture containing 50 mM Tris-HCl (pH 8.0), 1 mM NADP⁺, 10 mM MgCl₂, and 2 mM ATP. Absorbance was measured spectrophotometrically before and after the addition of 4 µg of purified hexokinase and glucose-6-phosphate dehydrogenase [35].

Lactate release into the culture medium was quantified by measuring the reduction of NAD⁺ at 340 nm. Reaction mixture contained 10 µL of culture medium, 100 mM Tris-HCl (pH 8.0), and 5 mM NAD⁺. Absorbance was recorded before and after the addition of 4 µg of purified lactate dehydrogenase (LDH) [35].

Data from both assays were normalized to cell number. In detail, at the end of incubation, cells were harvested, detached by trypsinization, and counted to normalize the measured concentration changes to the total number of cells per well. The values were then expressed per 10⁶ cells.

The efficiency of aerobic glycolysis was estimated by calculating the percentage of lactate actually released by ARPE-19 cells relative to the theoretical maximum lactate production, assuming that each molecule of glucose is completely converted to two molecules of lactate via glycolysis.

### Evaluation of substrate dependence of oxidative phosphorylation in RPE

To assess potential changes in the dependence of OxPhos on respiratory substrates (glutamine, fatty acids, and glucose) following incubation with POS or insulin treatment, ATP synthesis via the F₁F₀-ATP synthase (ATP synthase) and oxygen consumption rates (OCRs) were measured in ARPE-19 cells. Measurements were performed in the presence of 3 µM BPTES (a glutaminase inhibitor), 4 µM etomoxir (a CPT1-A inhibitor), or 2 µM UK5099 (a mitochondrial pyruvate carrier inhibitor) [36].

For each experiment, 2 × 10⁵ ARPE-19 cells suspended in growth medium were used. Cells were permeabilized for 1 min with 0.03 mg/mL digitonin before analysis. ATP synthesis was initiated by the addition of 0.1 mM ADP and monitored every 30 s for 2 min using a luciferin/luciferase chemiluminescence assay (GloMax^®^ 20/20 Luminometer, Promega, Milan, Italy). ATP standard solutions (10⁻⁸–10⁻⁵ M) were used for calibration (ATP Bioluminescence Assay Kit CLS II, Roche, Basel, Switzerland). Oxygen consumption was measured with an amperometric oxygen electrode (Unisense Microrespiration System, Unisense A/S, Aarhus, Denmark) in a closed chamber.

### Statistical analysis

Results are representative of at least 3 independent experiments. All data were analyzed with GraphPad Prism 9.0 software (GraphPad Software, San Diego, CA, USA). Data were expressed as the mean ± SD and then analyzed using *t-test* with Welch’s correction, one-way ANOVA followed by a Dunnett’s multiple comparison test, and two-way ANOVA followed by Šídák’s multiple comparisons test. Differences were considered statistically significant if the error probability was *p* < 0.05.

## Results

### ARPE-19 cells secrete insulin, and its release increased after rod OS phagocytosis

**Fig. 1:**
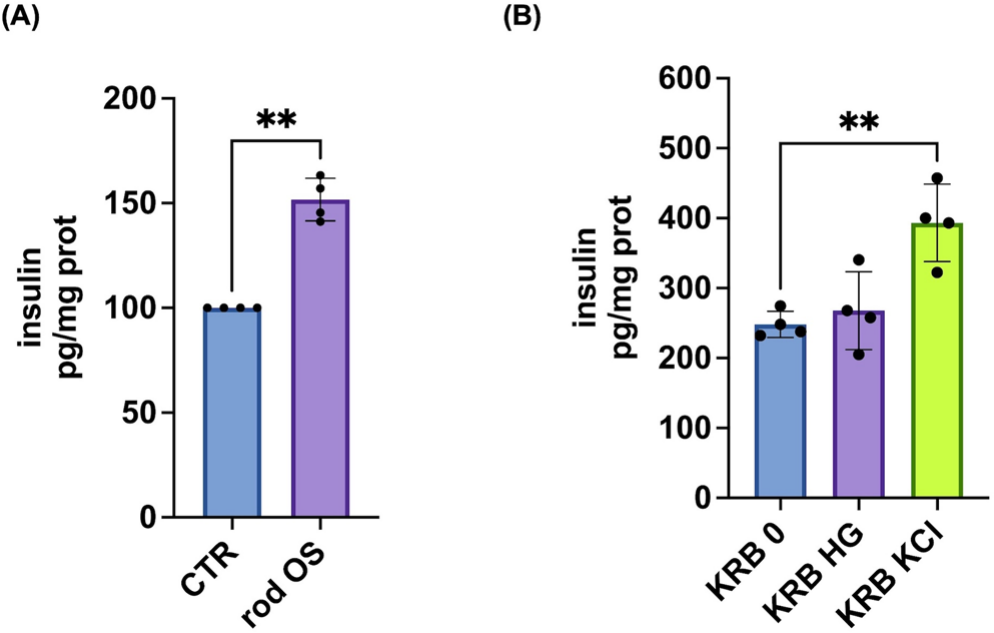
Insulin secretion in ARPE-19 cells. All data are obtained from: (i) ARPE-19 cells maintained in resting conditions (CTR) and after rod OS phagocytosis (rod OS) (**A**); (ii) ARPE-19 cells maintained in Kreb’s Ringer Bicarbonate buffer (KRB), and supplemented with 16.7 mM glucose (KRB HG) or 40 mM KCl (KRB KCl) (**B**). Each panel is representative of 4 independent experiments, and data are expressed as mean ± SD. Statistical analysis was performed by *t-test* with Welch’s correction for Panel A, and one-way ANOVA followed by a Dunnett’s multiple comparison test for Panel B. *p < 0.05, **p < 0.01

Since Etchegaray et al. have demonstrated an insulin release by RPE [24], we first verified whether ARPE-19 cells are capable of secreting insulin, performing secretion assays under different experimental conditions. In one set of experiments, we compared insulin release under physiological conditions and after rod OS phagocytosis. The results demonstrated that ARPE-19 cells already release insulin into the culture medium under basal conditions, which increased after the rod OS phagocytosis (Fig. 1A).

In addition, ARPE-19 cells were stimulated with KRB HG or KRB KCl, assessing insulin secretion in response to elevated glucose levels or a depolarizing stimulus, respectively. The results showed that while glucose did not affect insulin secretion, stimulation with KCl induced a significant increase in insulin release (Fig. 1B).

### ARPE-19 cells express pancreatic beta cell markers

To verify whether ARPE-19 cells express proteins involved in insulin production and processing, we analyzed the expression of some pancreatic beta-cell markers.

**Fig. 2:**
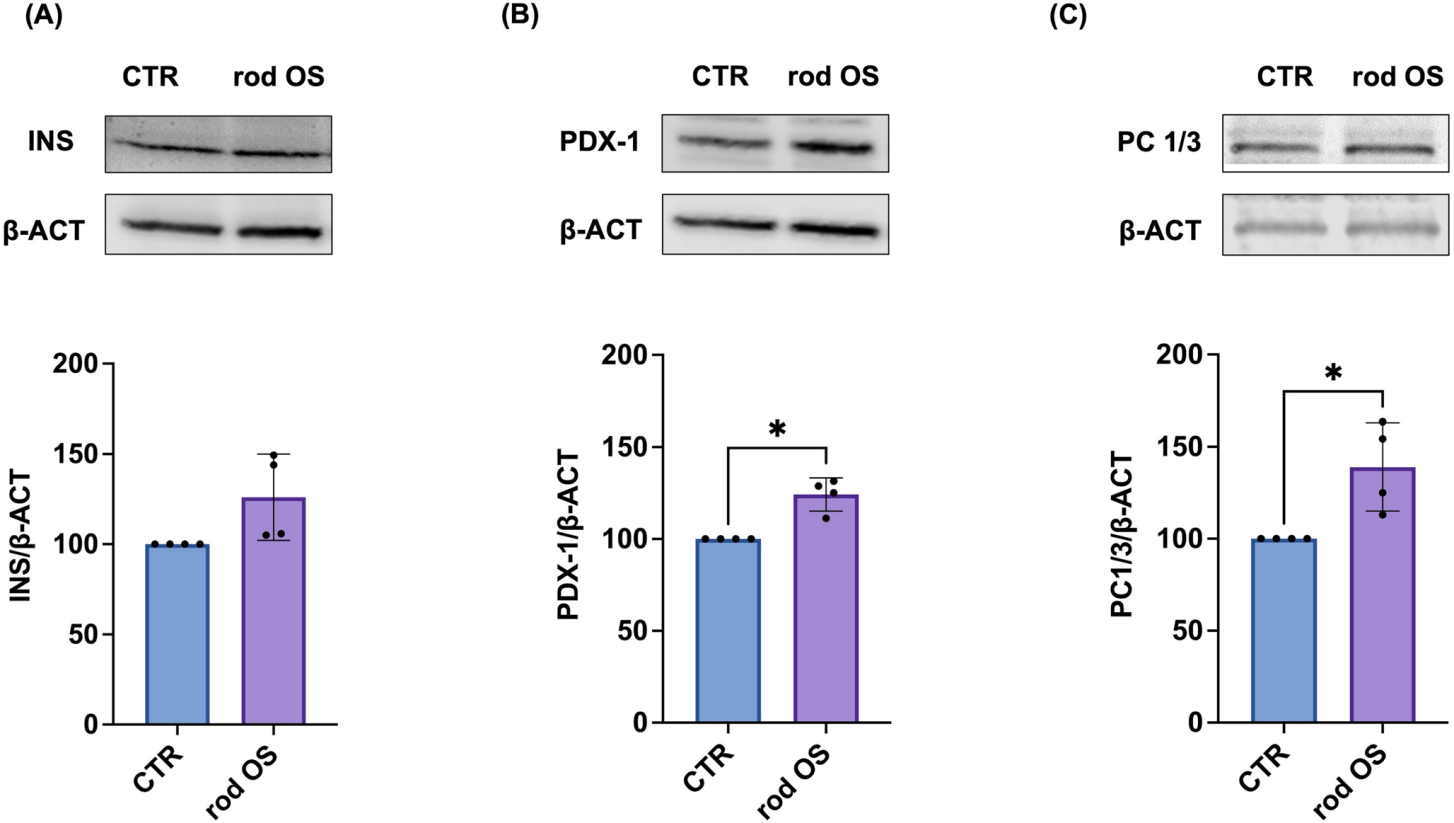
Expression of pancreatic beta-cell markers in ARPE-19 cells. All data are obtained from resting ARPE-19 cells (CTR) and from cells after rod OS phagocytosis (rod OS). Cells were lysed and immunoblotted with antibodies against (**A**) insulin (INS), (**B**) PDX-1, and (**C**) PC 1/3. Each panel reports a representative Western blotting signal and quantification of densitometries of Western blot bands normalized against β-Actin signal. The panel is representative of 4 independent experiments, and data are expressed as mean ± SD. Statistical analysis was performed by *t-test* with Welch’s correction. *p < 0.05

Firstly, data show that ARPE-19 cells expressed insulin protein, which did not change after rod OS phagocytosis (Fig. 2A). ARPE-19 also expressed PDX-1, a key transcription factor regulating proinsulin gene expression, which was upregulated after rod OS phagocytosis (Fig. 2B). In addition, we also investigated the expression of prohormone convertase 1/3 (PC 1/3), a crucial enzyme responsible for processing proinsulin into mature insulin. PC 1/3 is synthesized as a prohormone of 97 KDa, which is converted to active forms with a molecular weight ranging from 66 to 87 KDa [37, 38]. We observed that ARPE-19 cells express PC 1/3 and that its expression was significantly increased after rod OS phagocytosis (Fig. 2C).

**Fig. 3:**
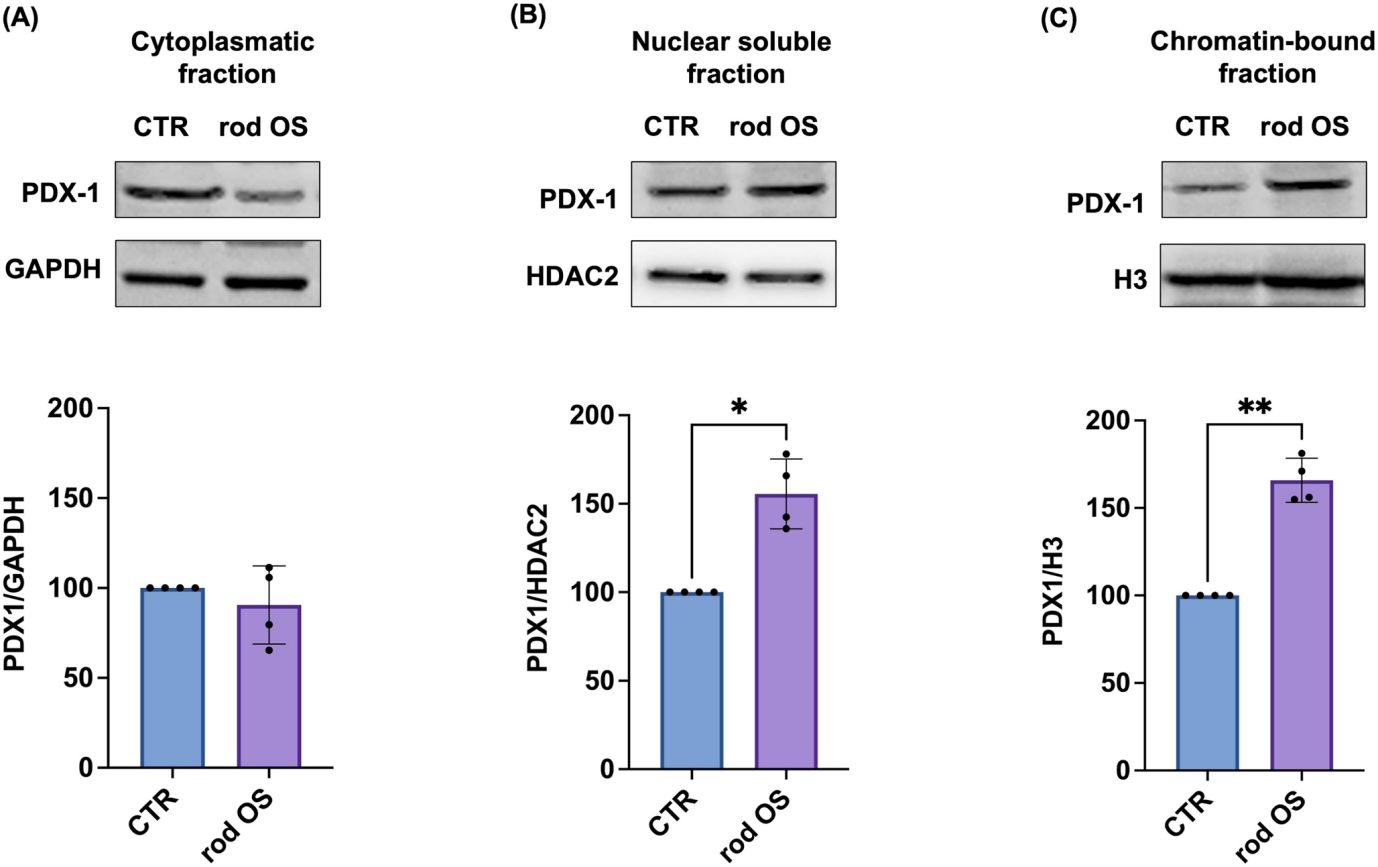
Effects of rod OS phagocytosis on intracellular distribution of PDX-1. All data are obtained from resting ARPE-19 cells (CTR) and from cells after rod OS phagocytosis (rod OS). PDX-1 expression in cytoplasmic fraction (**A**), nuclear soluble fraction (**B**), and chromatin-bound fraction (**C**). The same membrane was excised at the level of the molecular weight representing 30 kDa, and the fragments were immunoblotted, respectively, for PDX-1 (46 kDa) and H3 (17 kDa); then the upper part of the membrane was reblotted with GAPDH (37 kDa) and HDAC2 (60 kDa). Each panel reports a representative Western blotting signal and quantification of densitometries of Western blot bands normalized against a specific housekeeping protein for each fraction. All panels are representative of 4 individual experiments, and data are expressed as mean ± SD. Statistical analysis was performed by t-test with Welch’s correction. * p < 0.05, ** p < 0.01

To verify whether rod OS phagocytosis may activate PDX-1, we investigated its subcellular localization, observing that rod OS phagocytosis induced the translocation of PDX-1 into the nucleus and an enhancement of PDX-1-binding to the chromatin (Fig. 3).

### Activation of an autocrine insulin signalling pathway in ARPE-19 cells

**Fig. 4:**
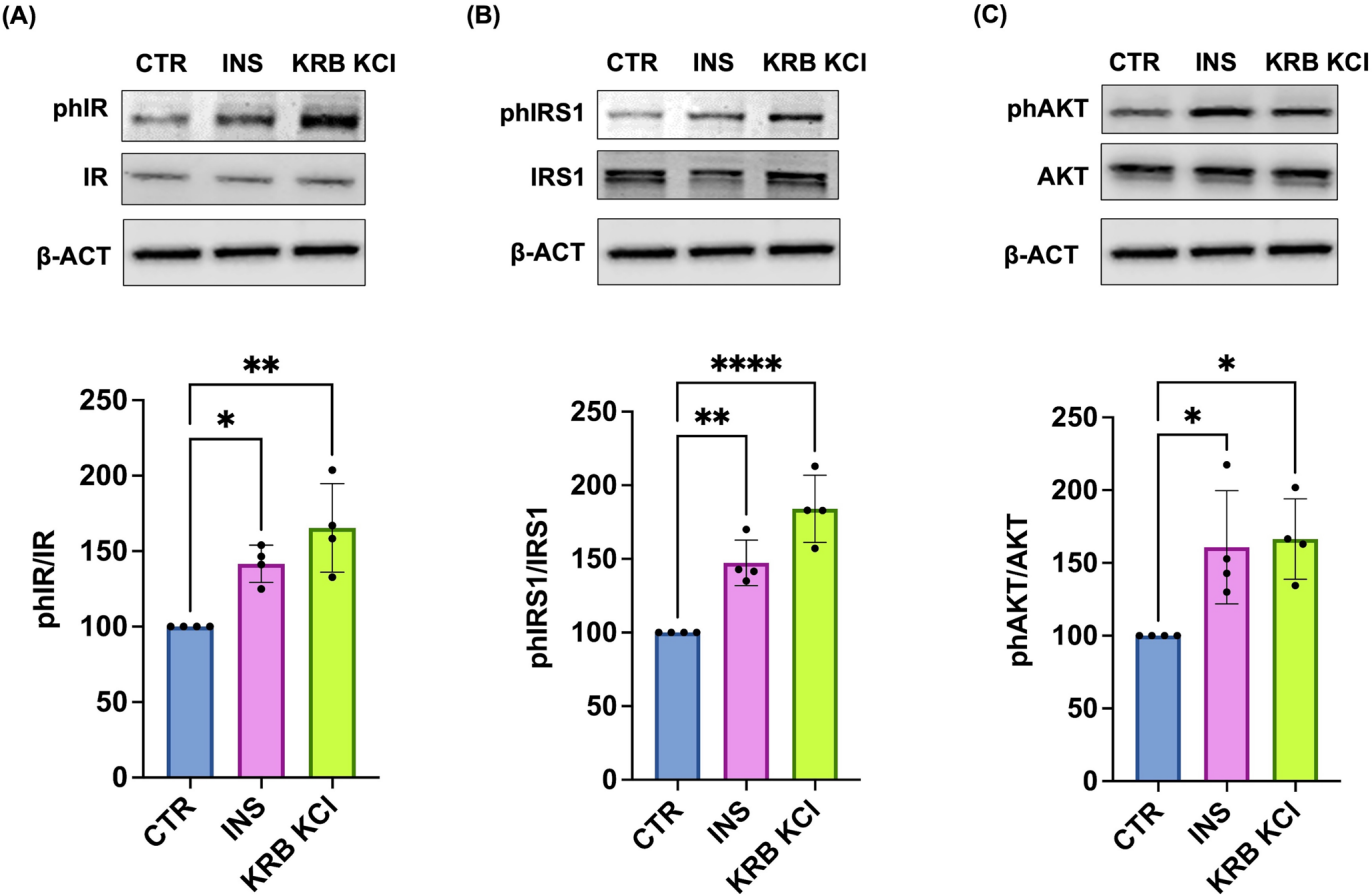
Autocrine signalling of insulin in ARPE-19 cells. All data are obtained from ARPE-19 cells serum-starved overnight and then exposed for 5 min to 100 nM insulin (INS) or KRB with 40 mmol/L of KCl (KCl). Then, cells were lysed and immunoblotted with antibodies against phIR and total IR (**A**), phIRS-1 and total IRS-1 (**B**), and phAKT and total AKT(**C**). Representative Western blotting and quantification of densitometries of Western blot bands are shown. Each panel reports a representative Western blotting signal and the quantification of the ratio between the phosphorylated and total protein, normalized against β-Actin signal. All panels are representative of 4 individual experiments, and data are expressed as mean ± SD. Statistical analysis was performed with one-way ANOVA followed by Dunnett’s multiple comparisons test. * p < 0.05, *** p < 0.001

To determine whether local insulin produced by the ARPE-19 can elicit an autocrine response, serum-starved ARPE-19 cells were stimulated with conditioned medium collected from ARPE-19 cells (containing auto-released insulin) previously exposed to KRB containing a high concentration of KCl. Activation of the insulin signalling pathway was assessed by measuring the phosphorylation of the IR, insulin receptor substrate-1 (IRS-1), and AKT in resting ARPE-19 cells. In parallel, a separate set of cells was treated with exogenous insulin as a positive control. The results showed that insulin secreted by ARPE-19 triggers canonical insulin signalling pathways, as indicated by a significant increase in the phosphorylation of IR (Fig. 4A) and its downstream substrates, IRS-1 (Fig. 4B) and AKT (Fig. 4C), which was comparable to that observed following insulin exposure.

**Fig. 5:**
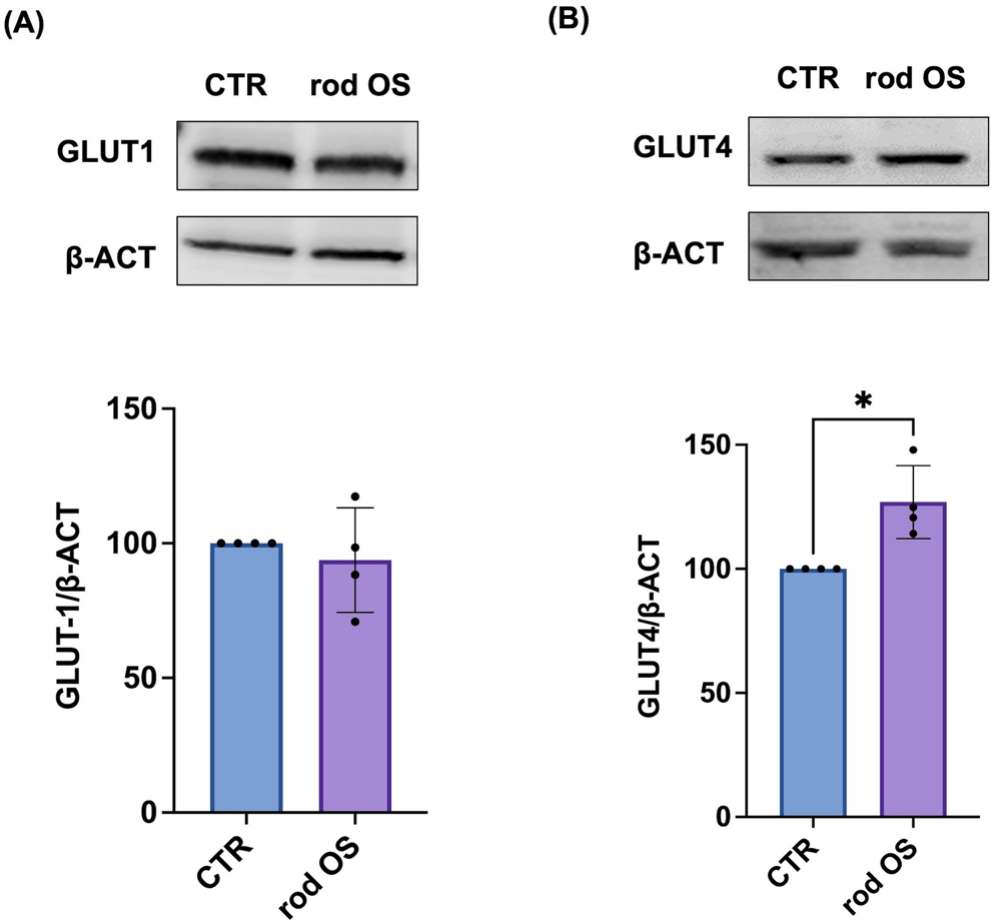
Expression of glucose transporters in ARPE-19 cells after rod OS phagocytosis. All data are obtained from resting ARPE-19 cells (CTR) and from cells after rod OS phagocytosis (rod OS). Cells were lysed and immunoblotted with an antibody against GLUT1 (**A**) and GLUT4 (**B**). Each panel reports a representative Western blotting signal and quantification of densitometries of Western blot bands normalized against β-Actin signal. All panels are representative of 4 individual experiments, and data are expressed as mean ± SD. Statistical analysis was performed by *t-test* with Welch’s correction. *p < 0.05

It is well known that RPE cells express GLUT1, the main glucose transporter in RPE cells. Moreover, in situ hybridization revealed the presence of the insulin-regulated glucose transporter GLUT4 in the retina [38]. Therefore, to assess the functional consequence of insulin pathway activation, we analyzed the expression of the constitutively expressed GLUT1 and of the insulin-inducible transporter GLUT4. While GLUT1 expression remained unchanged (Fig. 5A), GLUT4 levels increased following rod OS phagocytosis (Fig. 5B).

### Insulin pathway activation enhances glucose uptake and reduces lactate release in RPE cells

**Fig. 6:**
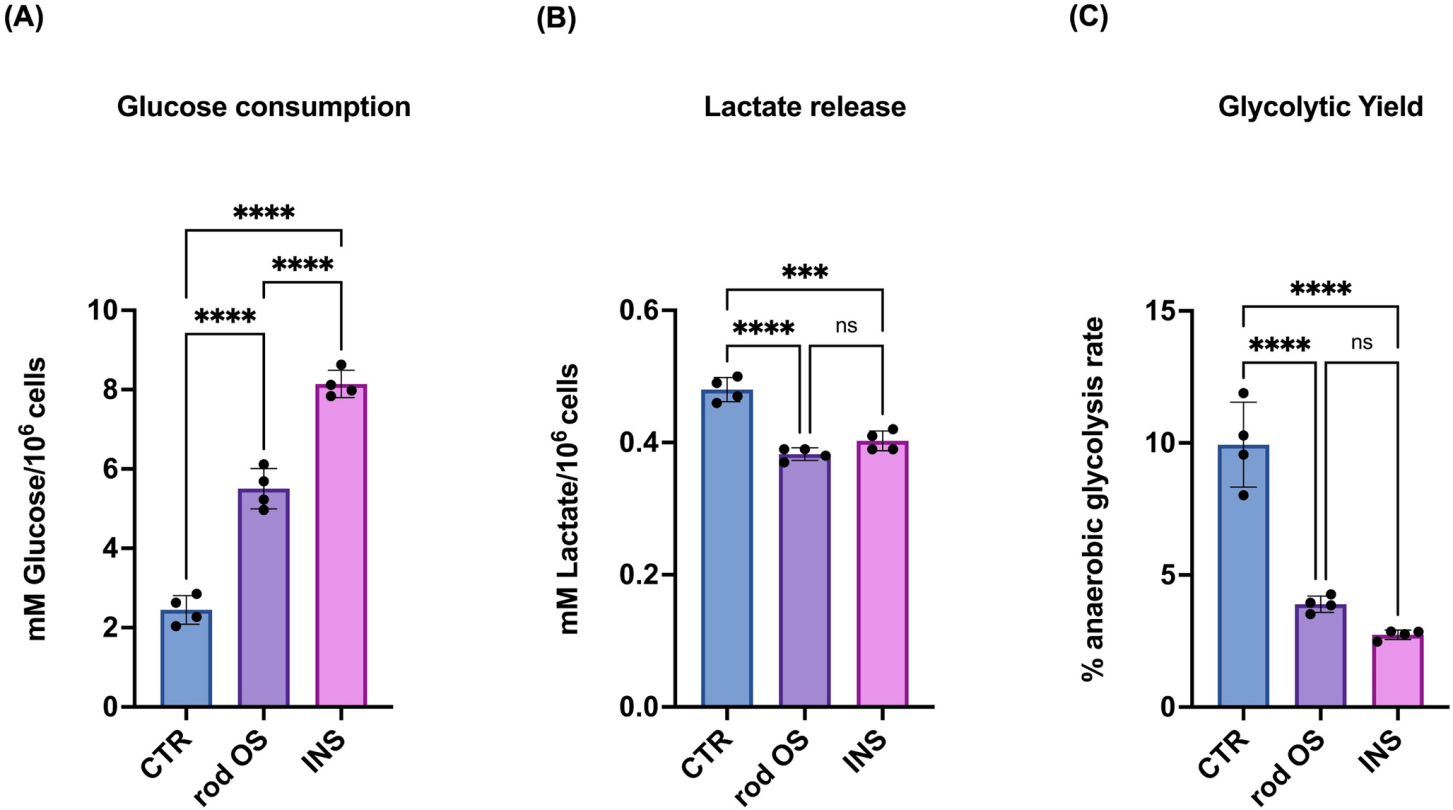
Glucose uptake, lactate release, and aerobic glycolysis yield in ARPE-19 cells after rod OS phagocytosis or insulin incubation. All data are obtained from growth media of resting ARPE-19 cells (CTR), from cells after rod OS phagocytosis (rod OS), and after insulin treatment (INS). Glucose consumption (**A**), lactate release (**B**), and aerobic glycolysis yield (**C**) have been reported. Each panel is representative of 4 individual experiments, and data are expressed as mean ± SD. Statistical analysis was performed by one-way ANOVA followed by Dunnett’s multiple comparisons test. ns: not significant, ***p < 0.001, ****p < 0.0001

Since one of the key metabolic actions of insulin is the stimulation of glucose uptake through the upregulation of glucose transporters, and our data indicated an increased expression of GLUT4, we assessed glucose consumption in ARPE-19 cells stimulated with insulin 48 h after rod OS phagocytosis. Data show that rod OS phagocytosis also resulted in a threefold increase in glucose consumption (Fig. 6A), a value similar to that obtained with exogenous insulin stimulation, which increased the glucose entering by about fourfold. Interestingly, this rise in glucose uptake was not accompanied by a corresponding increase in lactate release, which decreased following treatment with either insulin or rod OS phagocytosis (Fig. 6B). This discrepancy between glucose consumption and lactate production indicates a slowdown of aerobic glycolysis (Fig. 6C), suggesting that glucose catabolism induced by rod OS phagocytosis and insulin proceeds predominantly through OxPhos.

### Insulin pathway activation increased the OxPhos activity and its glucose-dependence in ARPE-19 cells

**Fig. 7:**
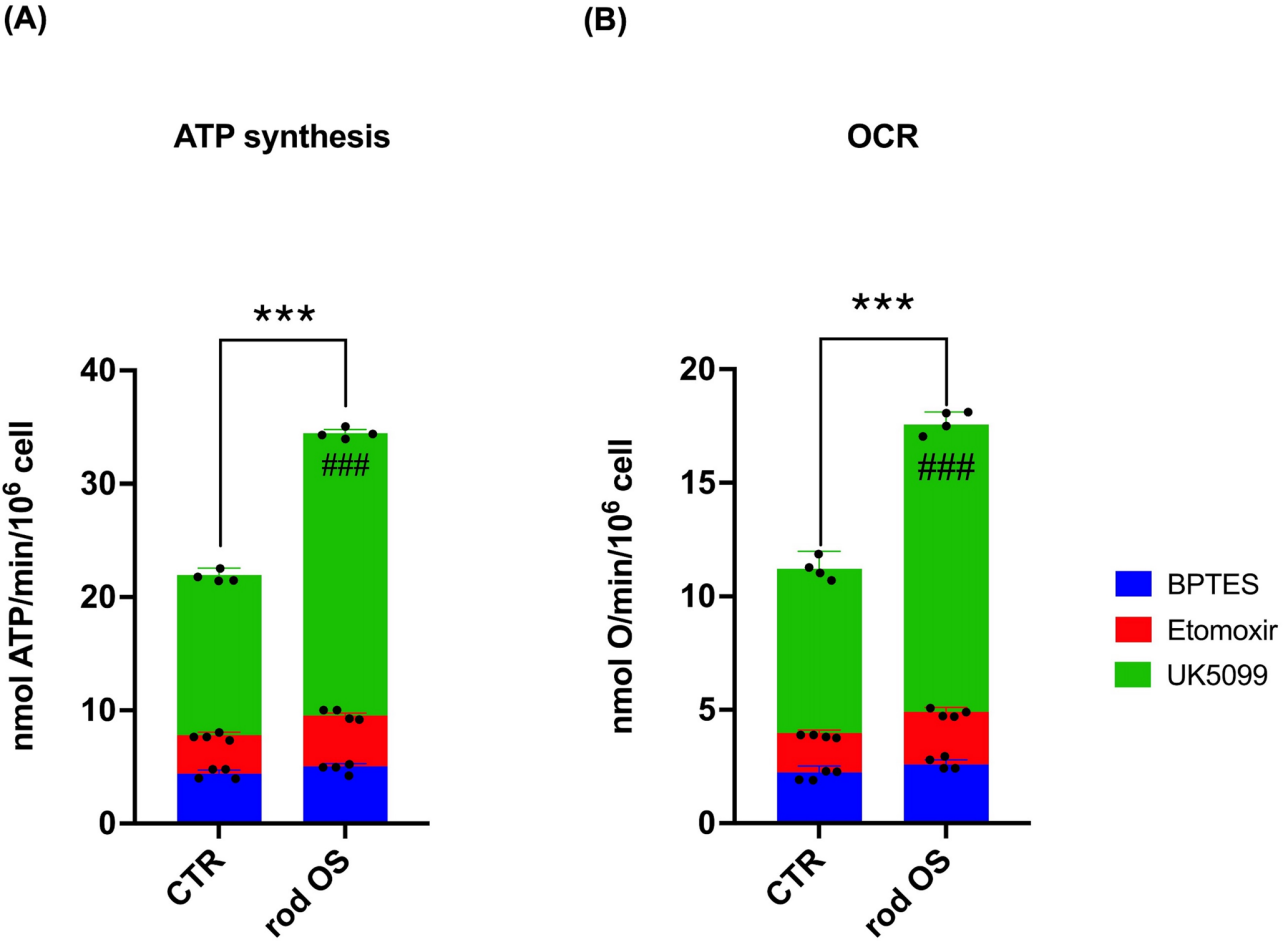
OxPhos metabolic dependence in ARPE-19 cells after rod OS phagocytosis. All data were obtained from resting ARPE-19 cells (CTR) and from cells after rod outer segment phagocytosis (rod OS). Experiments were performed in the presence of BPTES (a glutaminase inhibitor, indicating metabolic dependence on amino acids), etomoxir (an inhibitor of fatty acid transport into mitochondria, indicating metabolic dependence on fatty acids), and UK5099 (a pyruvate carrier inhibitor, indicating metabolic dependence on glucose oxidation), to assess the contribution of different energy substrates to OxPhos. ATP synthesis (**A**) and OCR (**B**) are shown. Each panel is representative of four independent experiments. Statistical analysis was performed using two-way ANOVA followed by Šídák’s multiple comparisons test. Each panel is representative of 4 individual experiments, and data are expressed as mean ± SD ***p < 0.001 versus CTR for total ATP synthesis and OCR after rod OS phagocytosis; ###*p* < 0.001 versus CTR for ATP synthesis or OCR inhibited by UK5099 (thus dependent on glucose oxidation) after rod OS phagocytosis

To verify the hypothesis that the insulin autocrine signal enhances OxPhos in ARPE-19 cells, ATP synthase activity and OCR were measured under basal conditions and following rod OS phagocytosis. As shown in Fig. 7, ARPE-19 cells exhibited a significant increase in both ATP synthesis (A) and OCR (B) after incubation with rod OS, indicating activation of oxidative metabolism. Since these metabolic parameters were assessed in the presence of BPTES, etomoxir, and UK5099—specific inhibitors of glutamine, fatty acid, and glucose oxidation, respectively— we also observed that the increase in ATP production and respiration was entirely dependent on glucose since the ATP synthase activity and OCR sensitive to BPTES and etomoxir remained similar after the rod OS phagocytosis to the control. These findings suggest that the additional glucose taken up by ARPE-19 cells after rod OS phagocytosis is metabolized predominantly through OxPhos.

## Discussion

This study demonstrates that ARPE-19 cells can produce and release insulin, which appears to act as an autocrine signal, potentially contributing to the regulation of glucose metabolism.

In detail, our data show that ARPE-19 cells secrete insulin in response to depolarizing stimuli, but not to high-glucose exposure, suggesting a functional behavior distinct from that of pancreatic β-cells. However, RPE cells and human retina express the receptor for the incretin hormone glucagon-like peptide-1 (GLP-1). The co-expression of GLP-1 receptors and the ability to synthesize and secrete insulin suggest that RPE cells share functional similarities with pancreatic β-cells, at least in part [39]. Specifically, ARPE-19 cells seem to maintain components of β-cells molecular machinery involved in insulin biosynthesis and processing, as they express both PDX-1 and PC1/3, two key proteins implicated in insulin production [40, 41]. Interestingly, the expression of these markers increases in ARPE-19 cells following rod OS phagocytosis, suggesting that insulin synthesis and processing might be stimulated by one of the principal physiological functions of the RPE. Despite this, the intracellular insulin concentration in phagocytosing ARPE-19 cells appears comparable to that of resting cells. This apparent discrepancy may be explained by the enhanced insulin secretion observed following rod OS phagocytosis. In addition, insulin secreted by ARPE-19 cells activated the canonical insulin signalling pathway in an autocrine manner, as indicated by increased phosphorylation of IR, IRS-1, and AKT. Nonetheless, local retinal insulin acts in a microenvironment which is much more sensitive than the systemic circulation. The activation pattern of IR and its downstream targets appeared similar to that induced by insulin, confirming that the pathway stimulated by insulin endogenously released from ARPE-19 cells responds to KCl-induced depolarization. Consistent with this, GLUT4 expression was upregulated, potentially contributing to the enhanced glucose uptake associated with insulin signalling.

This self-stimulation suggests the presence of an autocrine signalling mechanism triggered by rod OS phagocytosis, probably to support this highly energy-demanding process [24]. Although literature reports that RPE transfers most choroidal glucose to photoreceptors to sustain phototransduction [3, 8, 42], it is possible to hypothesize that, during phagocytosis, RPE cells may utilize a portion of this glucose to meet their increased energy demands. Specifically, our data demonstrate that RPE selectively increases pyruvate-supported OxPhos in response to rod OS phagocytosis, while the oxidative metabolism relying on glutamine and fatty acids remains unaffected. This suggests that glucose, internalized by ARPE-19 cells following insulin autocrine signalling, could be converted to pyruvate via glycolysis, subsequently fueling mitochondrial ATP synthesis. Interestingly, alongside the increased OxPhos, a concomitant reduction in aerobic glycolysis and lactate release was observed. This shift could reflect the higher energy demand associated with phagocytosis, as the oxidation of one glucose molecule through OxPhos yields approximately 36 ATP, compared to only 2 ATP produced by aerobic glycolysis. On the other hand, RPE cells support their energy demands primarily through OxPhos rather than glycolysis, which is supported by the presence of abundant mitochondria [10].

Despite the observed autocrine effect, insulin exerts multiple actions on RPE. Insulin promotes RPE proliferation, likely via ERK1/2 pathway activation [43], enhances taurine uptake essential for retinal function [44], and stimulates endocytosis, reinforcing the RPE’s role in nutrient recycling and photoreceptor support [22]. At the molecular level, insulin upregulates the IR β subunit and modulates proteins implicated in retinal pathophysiology [22]. For example, insulin downregulates VEGF-A and angiotensinogen [41], suggesting its involvement in vascular regulation and extracellular matrix remodelling. The retina expresses both neuronal and vascular insulin receptors.

In light of these observations, RPE insulin production may play a crucial role in the prevention and modulation of retinal diseases, such as diabetic retinopathy, where chronic insulin resistance could impair RPE responsiveness and exacerbate disease progression [42]. For instance, Tarchick et al. (2019) demonstrated that RPE-specific IR knockout mice exhibit reduced a and b-wave amplitudes in electroretinogram recordings, indicative of compromised rod photoreceptor activity [22]. The downregulation of AKT in diabetes causes retinal cells apoptosis, impaired mitochondrial function, and oxidative stress production [45]. Moreover, since diabetic or oxidative stress conditions can alter the RPE and POS interaction [3], local insulin may contribute to stabilizing these interactions by sustaining AKT signalling, reducing oxidative stress, and promoting efficient metabolic coupling between the RPE and photoreceptors, ultimately supporting neuronal survival and photoreceptor function [46]. By acting in a microenvironment that is more sensitive than the systemic circulation, the local RPE insulin production may play a neuroprotective role mediated by AKT signalling, promoting neuronal survival, regulating oxidative stress, and vascular permeability.

However, it is essential to note that the employed in vitro system, consisting of only ARPE-19 cells, represents a limitation of the study to evaluate the effect of local insulin release on the retina. Although ARPE-19 cells are widely employed and retain several key features of RPE, including phagocytic activity and expression of metabolic enzymes, they do not fully recapitulate the phenotype of native, fully differentiated RPE, and the complex interaction with the other retinal cells. In detail, ARPE-19 lacks the full complexity of the retinal environment, including the continuous flux of metabolites that critically shape RPE metabolism in vivo. For example, Swarup et al. demonstrated that reducing GLUT1-mediated glucose transport across the murine RPE leads to photoreceptor degeneration and Müller glial activation, highlighting the essential role of RPE-controlled glucose supply for retinal homeostasis [9]. Hass et al. showed in vivo that photoreceptor-derived lactate fuels RPE metabolism, highlighting a tightly coupled metabolic ecosystem that cannot be fully recapitulated in isolated cell models [13]. These findings underscore that RPE metabolism is tightly integrated within a multicellular network. However, based on the same assumptions, it can also be hypothesized that the insulin released by ARPE-19 cells not only exerts an autocrine effect but may also activate insulin signalling in other cell types that exhibit high expression of the IR, such as photoreceptors. In type 2 diabetes, insulin resistance and signalling impairment compromise retinal neuroprotective pathways such as PI3K/Akt, promoting inflammation and vascular stress, ultimately causing diabetic retinopathy. Although our data may provide evidence of a potential intrinsic capability of RPE cells to produce insulin and activate autocrine signalling, consistent with in vivo observations of local insulin production [24], further studies using primary RPE cells, retinal organoids, or animal models will be necessary to determine the extent to which RPE-derived insulin contributes to retinal metabolism and photoreceptor support under physiological and pathological conditions.

## Conclusions

Our data support the idea that RPE could play a pivotal role as a regulator capable of modulating its own energy metabolism through locally produced insulin. Increasing RPE insulin production may locally compensate for the retinal deficit in insulin signalling. The intrinsic autocrine insulin signalling may represent an adaptive mechanism to sustain the high energetic demands of phagocytosis and retinal maintenance [25]. Understanding and harnessing this endogenous insulin pathway could open new therapeutic avenues for retinal neurodegenerative and metabolic diseases, particularly those associated with diabetes and oxidative stress.

## Supplementary Information

Below is the link to the electronic supplementary material.

Supplementary Material 1 To confirm that the adopted culture protocol—described in the Cell Line and Culture Conditions section of Materials and Methods—induces proper differentiation of ARPE-19 cells toward an epithelial phenotype, morphological changes in confluent ARPE-19 cells before (T0) and after 9 days (T9) of culture in medium containing 3% FBS were analyzed. Images in (A) show a transition from an elongated cell shape to a more cuboidal morphology, with cells more tightly adherent to one another. The lower panels represent magnifications of the areas outlined by black dashed lines in the corresponding upper panels. In addition, (B) and (C) report a representative Western blot signal and the corresponding densitometric analysis, normalized to actin, of ZO-1 and RPE65—two markers typical of differentiated RPE cells [49, 50]—showing an increase after 9 days of culture. All panels are representative of four independent experiments, and data in panels (B) and (C) are expressed as mean ± SD. Statistical analysis was performed using a t-test with Welch’s correction. **p < 0.01.

Supplementary Material 2 To verify the ability of ARPE-19 cells to internalize rod OS, cells were collected after 4 h of incubation with photoreceptors, extensively washed to remove non-internalized material, and analyzed by Western blot to assess the presence of rhodopsin, a marker of rod OS. (A) Western blot showing rhodopsin and actin (housekeeping) signals in the presence (+) or absence (−) of rod OS. (B) Densitometric analysis of rhodopsin normalized to actin. Data are representative of 4 independent experiments, and in (B) are reported as mean ± SD. Statistical analysis was performed using a t-test with Welch’s correction. ***p < 0.001.

Supplementary Material 3 The figure shows the expression of GAPDH, H2AC2, and H3 as markers of the cytoplasmic, nuclear soluble, and chromatin-bound fractions, respectively, evaluated by Western blot to assess the quality of subfractionation. Data are representative of four independent experiments.

Supplementary Material 4

## Data Availability

Further information and requests for resources and reagents should be directed to and will be fulfilled by the lead contact, Prof. Silvia Ravera (silvia.ravera@unige.it). All data supporting the conclusions of this study can be found in the Article.
