# Supplementary figures and images for: Insulin production in the retina drives autocrine signalling and metabolism reprogramming of the ARPE-19, a retinal pigment epithelium cellular model
Source: Cell Mol Life Sci. 2026 May 1;83(1):259. doi: 10.1007/s00018-026-06222-0 (PMC13287518; doi:10.1007/s00018-026-06222-0)

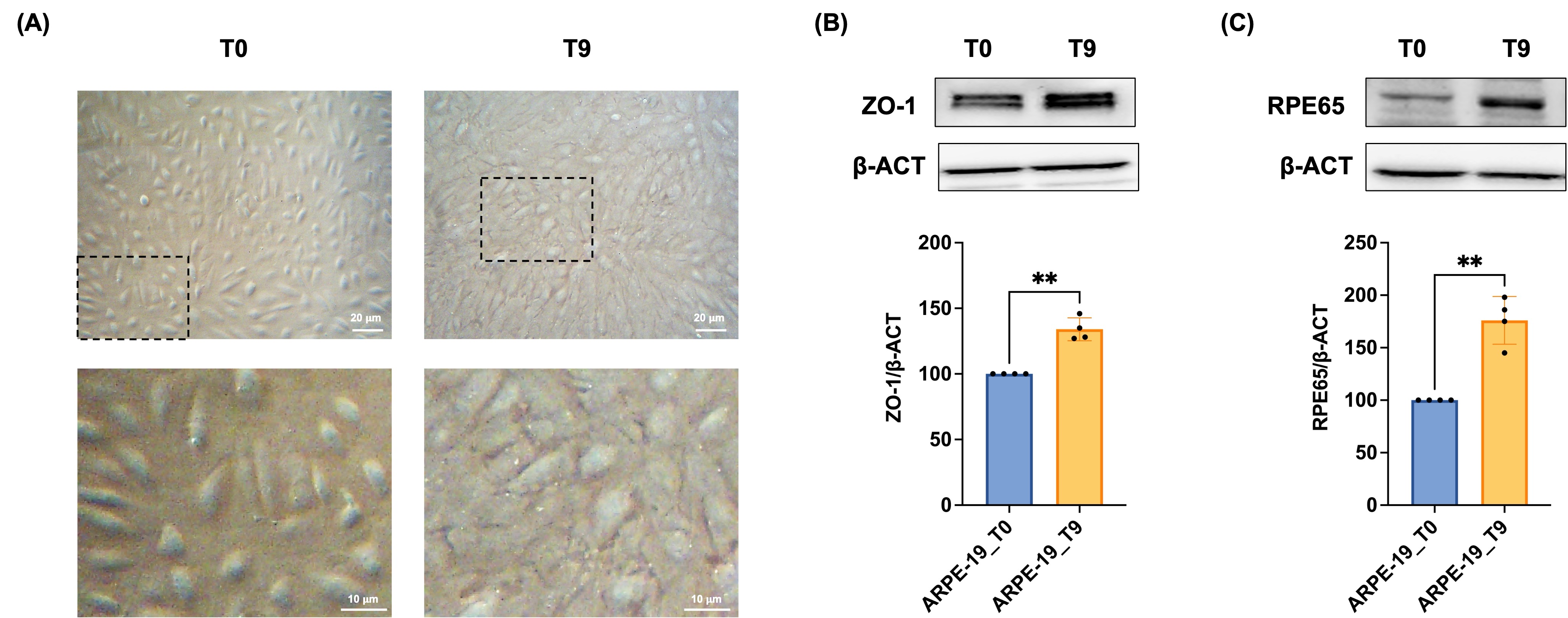

Supplement: Supplementary file 1 — Supplementary Material 1 To confirm that the adopted culture protocol—described in the Cell Line and Culture Conditions section of Materials and Methods—induces proper differentiation of ARPE-19 cells toward an epithelial phenotype, morphological changes in confluent ARPE-19 cells before (T0) and after 9 days (T9) of culture in medium containing 3% FBS were analyzed. Images in (A) show a transition from an elongated cell shape to a more cuboidal morphology, with cells more tightly adherent to one another. The lower panels represent magnifications of the areas outlined by black dashed lines in the corresponding upper panels. In addition, (B) and (C) report a representative Western blot signal and the corresponding densitometric analysis, normalized to actin, of ZO-1 and RPE65—two markers typical of differentiated RPE cells [49, 50]—showing an increase after 9 days of culture. All panels are representative of four independent experiments, and data in panels (B) and (C) are expressed as mean ± SD. Statistical analysis was performed using a t-test with Welch’s correction. **p < 0.01. [file 18_2026_6222_MOESM1_ESM.jpg]

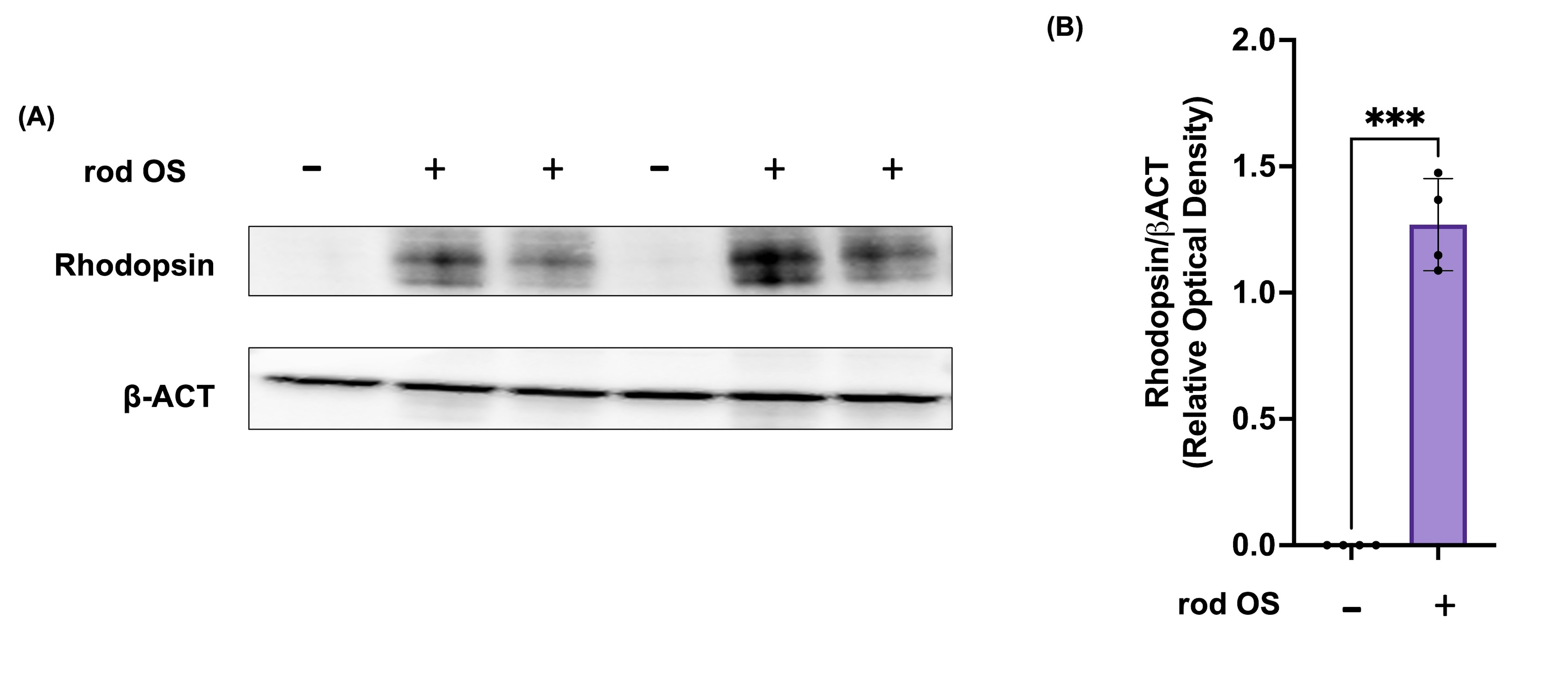

Supplement: Supplementary file 2 — Supplementary Material 2 To verify the ability of ARPE-19 cells to internalize rod OS, cells were collected after 4 h of incubation with photoreceptors, extensively washed to remove non-internalized material, and analyzed by Western blot to assess the presence of rhodopsin, a marker of rod OS. (A) Western blot showing rhodopsin and actin (housekeeping) signals in the presence (+) or absence (−) of rod OS. (B) Densitometric analysis of rhodopsin normalized to actin. Data are representative of 4 independent experiments, and in (B) are reported as mean ± SD. Statistical analysis was performed using a t-test with Welch’s correction. ***p < 0.001. [file 18_2026_6222_MOESM2_ESM.jpg]

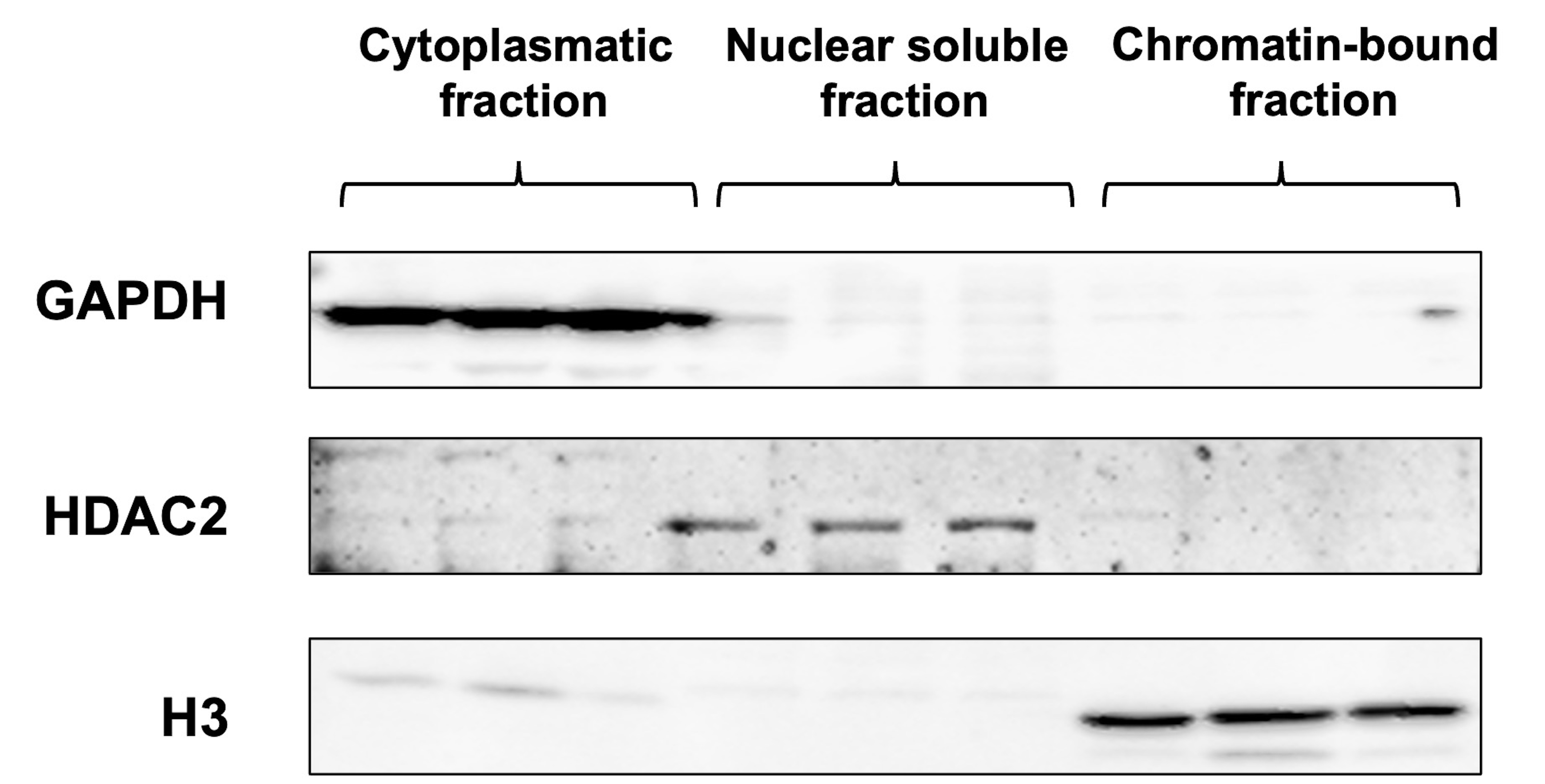

Supplement: Supplementary file 3 — Supplementary Material 3 The figure shows the expression of GAPDH, H2AC2, and H3 as markers of the cytoplasmic, nuclear soluble, and chromatin-bound fractions, respectively, evaluated by Western blot to assess the quality of subfractionation. Data are representative of four independent experiments. [file 18_2026_6222_MOESM3_ESM.jpg]
