# Supplementary material for: Insulin production in the retina drives autocrine signalling and metabolism reprogramming of the ARPE-19, a retinal pigment epithelium cellular model
Source: Cell Mol Life Sci. 2026 May 1;83(1):259. doi: 10.1007/s00018-026-06222-0 (PMC13287518; doi:10.1007/s00018-026-06222-0)

Figure 2

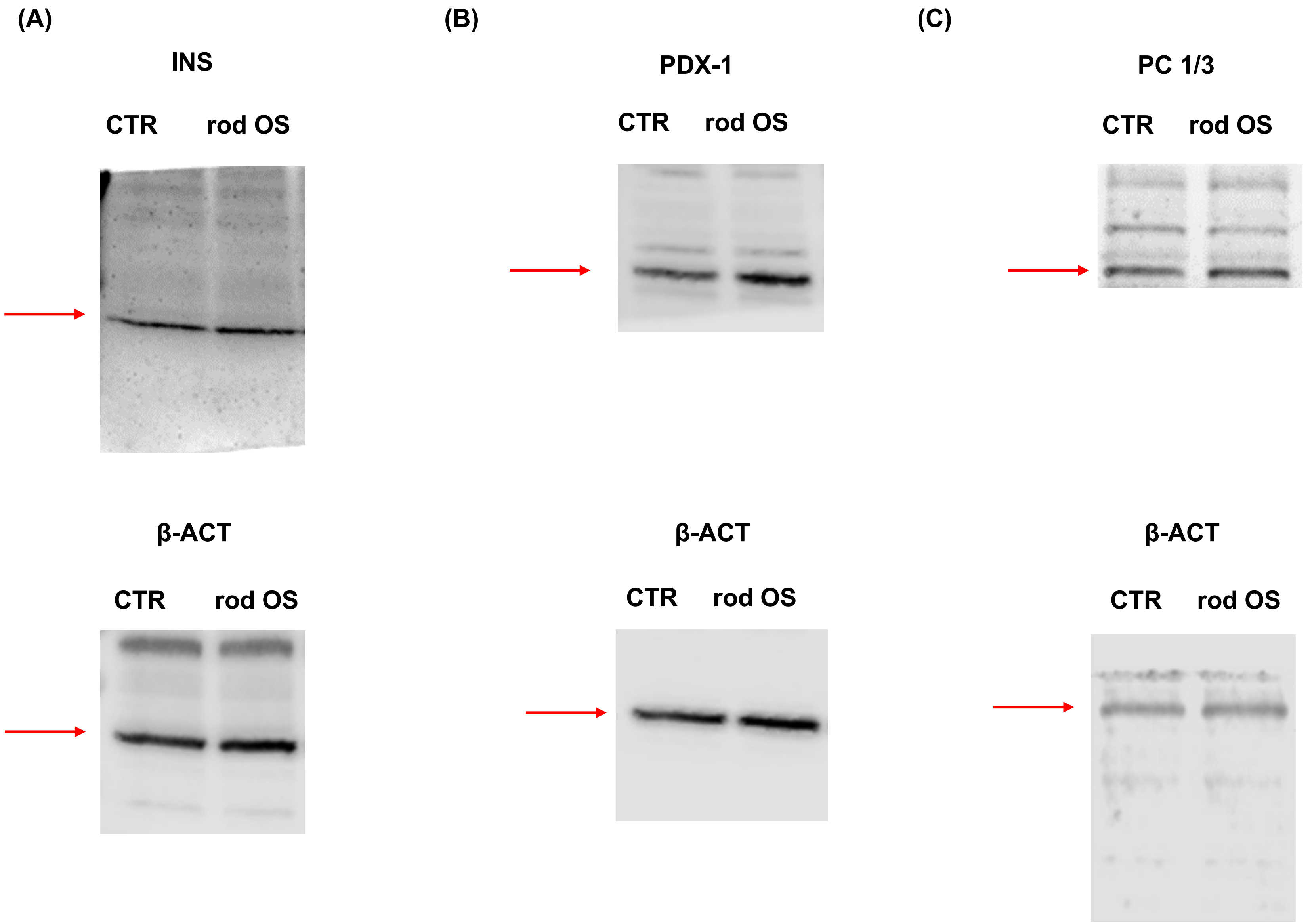

(A)
INS
CTR
rod OS
β-ACT
CTR
rod OS
(B)
PDX-1
CTR
rod OS
β-ACT
CTR
rod OS
(C)
PC 1/3
CTR
rod OS
β-ACT
CTR
rod OS

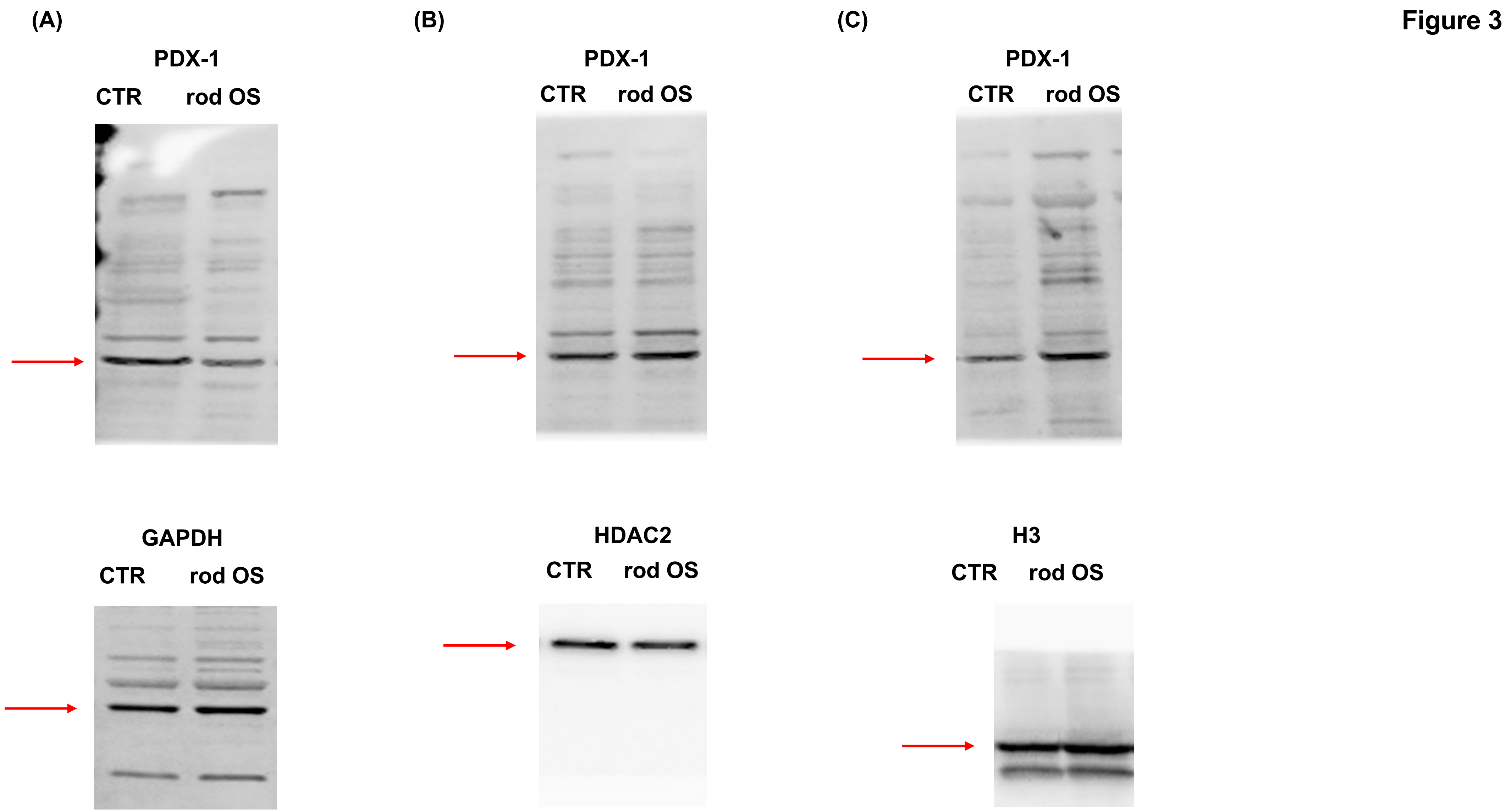
Figure 3
(A)
PDX-1
CTR
rod OS
GAPDH
CTR
rod OS
(B)
PDX-1
CTR
rod OS
HDAC2
CTR
rod OS
(C)
PDX-1
CTR
rod OS
H3
CTR
rod OS

Figure 4

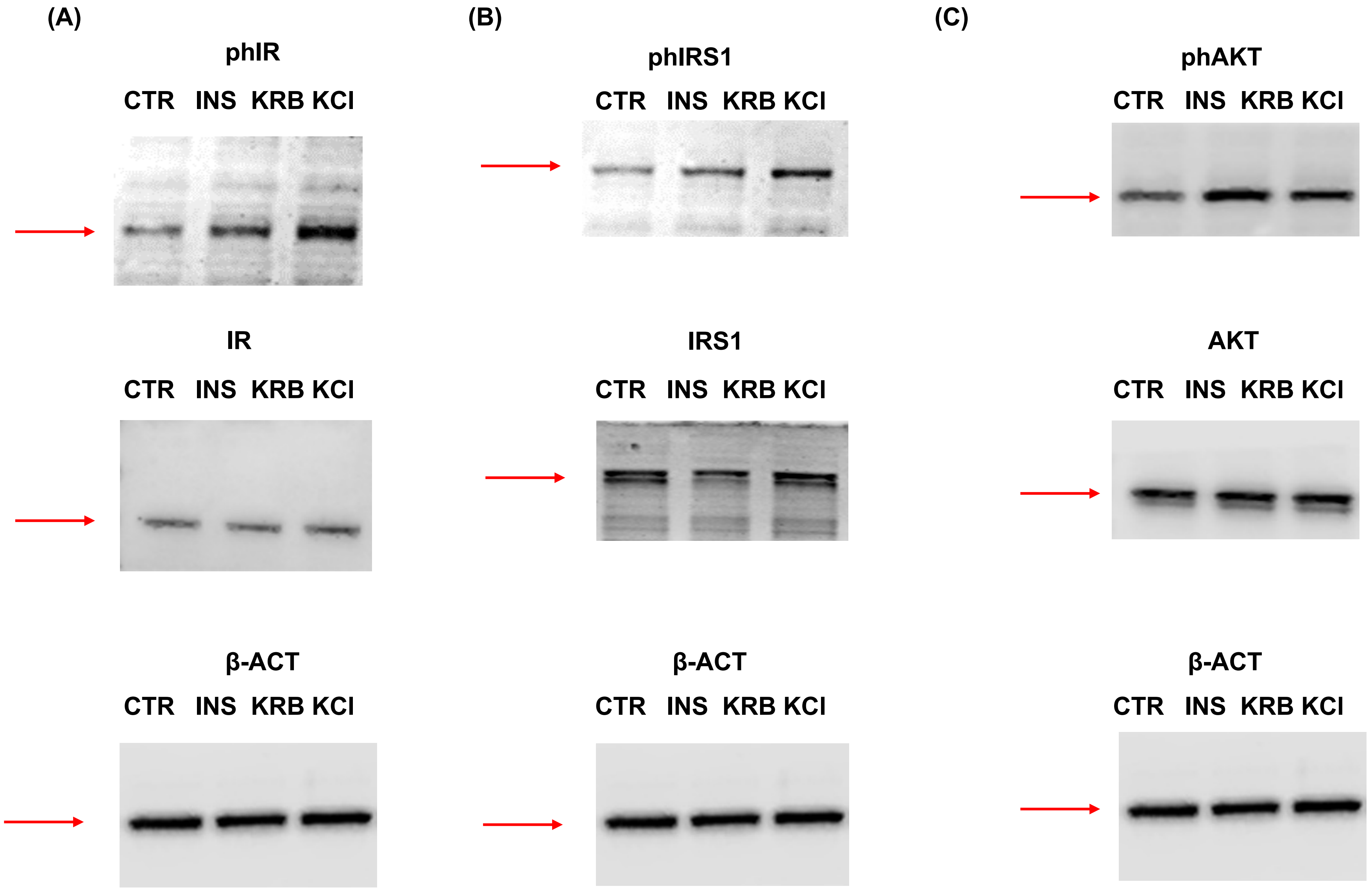
(A)
phIR
CTR INS KRB KCl
IR
CTR INS KRB KCl
β-ACT
CTR INS KRB KCl
(B)
phIRS1
CTR INS KRB KCl
IRS1
CTR INS KRB KCl
β-ACT
CTR INS KRB KCl
(C)
phAKT
CTR INS KRB KCl
AKT
CTR INS KRB KCl
β-ACT
CTR INS KRB KCl

Figure 5

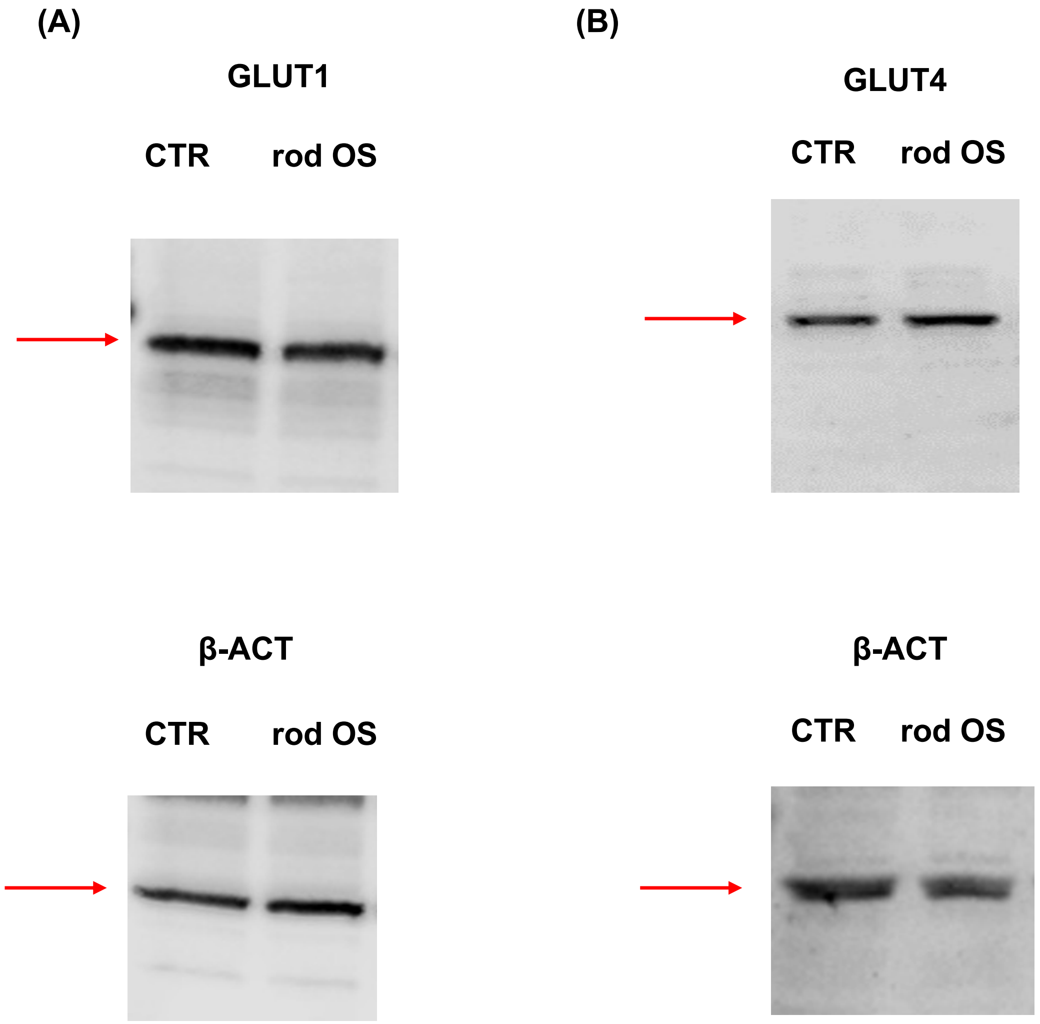
(A)
GLUT1
CTR
rod OS
β-ACT
CTR
rod OS
(B)
GLUT4
CTR
rod OS
β-ACT
CTR
rod OS

Suppl Figure 1

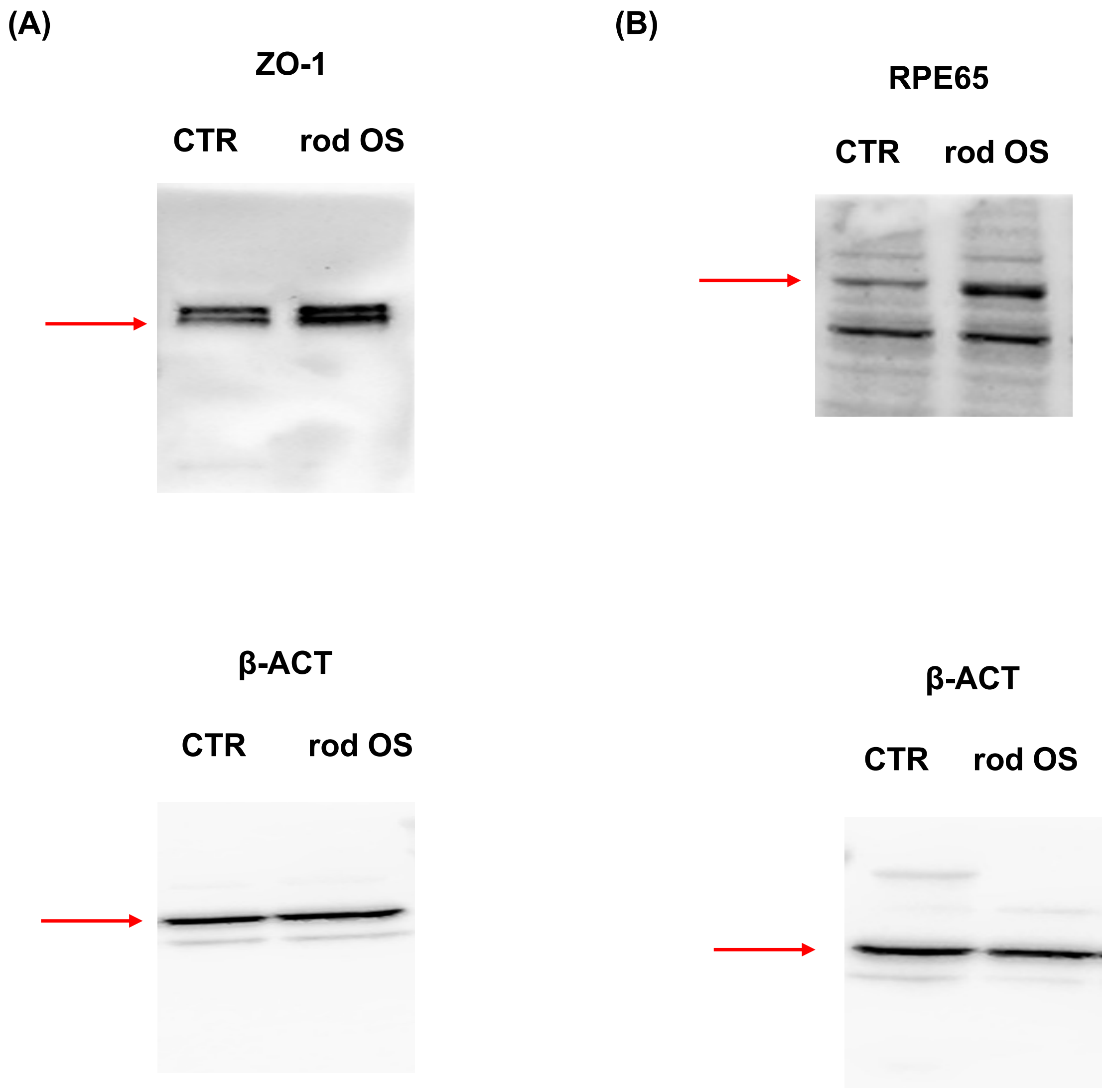

Suppl Figure 2

(A)

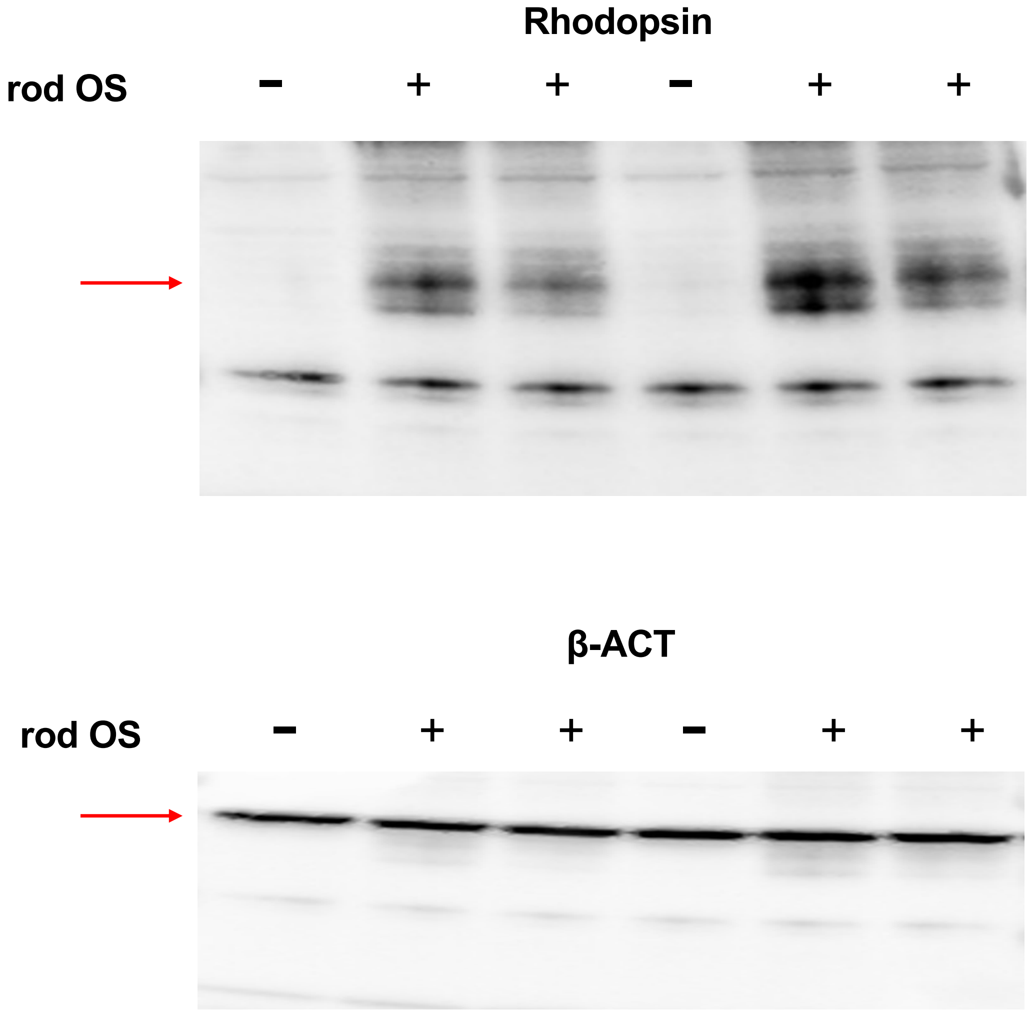

Suppl Figure 3

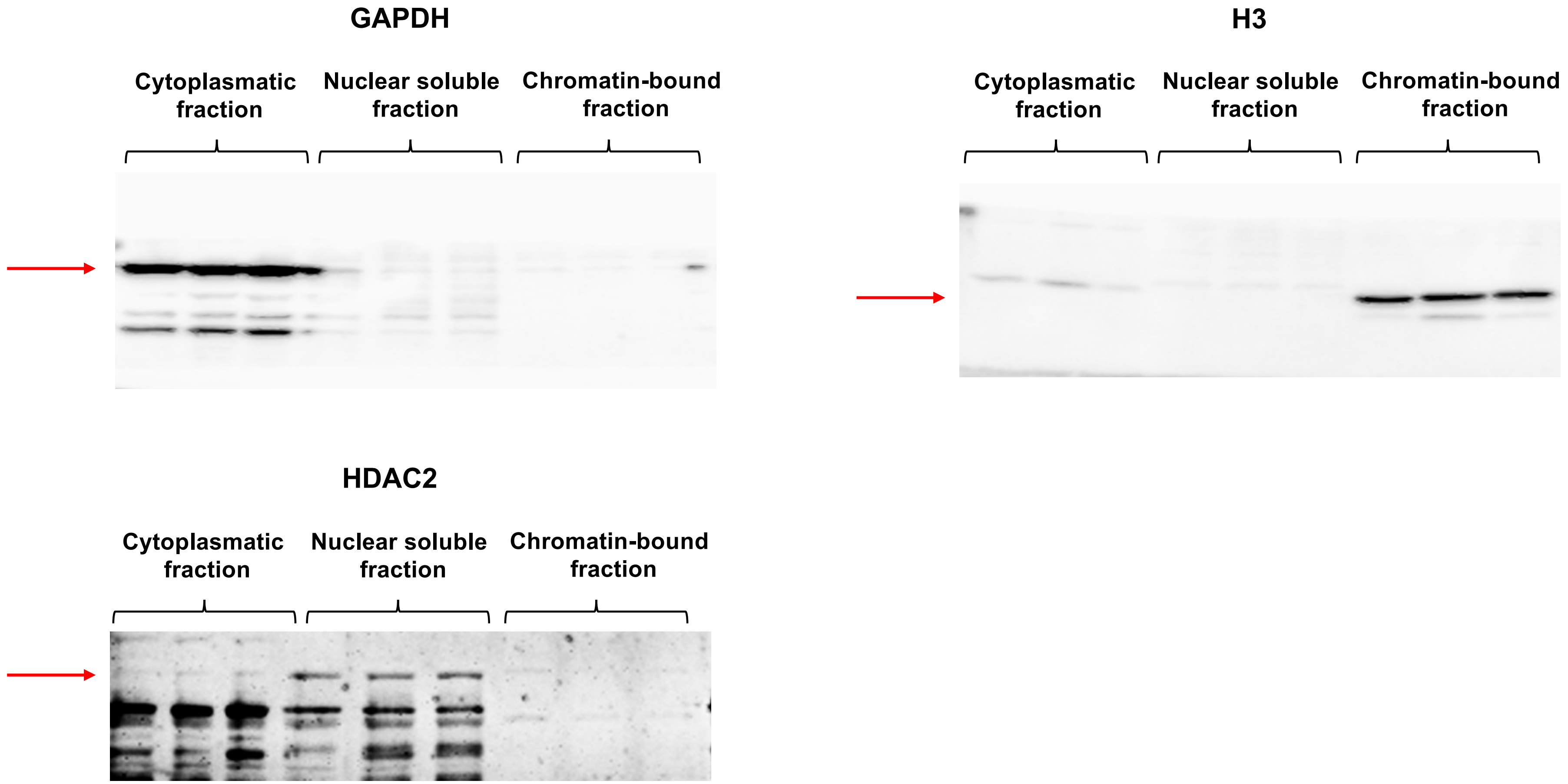

Supplement: Supplementary file 4 — Supplementary Material 4 [file 18_2026_6222_MOESM4_ESM.pdf]
